# Construction of risk prediction model for dysphagia in hospitalized elderly patients with frailty

**DOI:** 10.3389/fmed.2025.1659438

**Published:** 2025-08-14

**Authors:** Yuzhu Lin, Chen Xu, Xue Song, Qin Tan, Xiaolei Lian

**Affiliations:** Mianyang Central Hospital, School of Medicine, University of Electronic Science and Technology of China, Mianyang, China

**Keywords:** dysphagia, frailty, elderly, influencing factors, risk prediction model

## Abstract

**Background:**

Dysphagia is a common complication in elderly patients with frailty, affecting their prognosis and quality of life. Constructing a risk prediction model can help with early screening and intervention.

**Objective:**

To investigate the current status of dysphagia in hospitalized elderly patients with frailty, analyze its influencing factors, and construct a risk prediction model for dysphagia in hospitalized elderly patients with frailty.

**Methods:**

A total of 300 hospitalized elderly patients with frailty were selected as research subjects using a convenience sampling method from May to December 2024 in a tertiary general hospital in Mianyang. The survey tools included the General Information Questionnaire, Mini Nutritional Assessment-Short Form (MNA-SF), Geriatric Self-Efficacy Scale for Oral Health (GSEOH), Geriatric Oral Health Assessment Index (GOHAI), and 5-Item Geriatric Depression Scale (GDS-15). Data were analyzed using SPSS 26.0 software, and variable selection was conducted using the backward LR method to construct the risk prediction model.

**Results:**

A total of 300 questionnaires were distributed, and 287 valid questionnaires were retrieved, with an effective recovery rate of 95.7%. Among the 287 patients, 103 cases (35.9%) were identified with dysphagia. Among the 202 patients with a history of choking, 80 cases (39.6%) were identified as having swallowing disorders. In contrast, among the 85 patients without a history of choking, 23 cases (27.1%) had swallowing disorders. The difference was statistically significant (*χ*^2^ = 4.092, *p* = 0.043). Logistic regression analysis showed that age, history of coughing, polypharmacy, malnutrition, oral health-related self-efficacy, and oral health assessment index were risk factors for dysphagia in elderly patients with frailty (*p* < 0.05). The constructed risk prediction model was: Logit *p* = 0.770 × Age + 0.919 × Polypharmacy + 1.009 × History of Coughing + 1.208 × Malnutrition − 0.113 × Oral Health-Related Self-Efficacy − 0.262 × Oral Health Assessment Index + 10.200. The Hosmer–Lemeshow goodness-of-fit test indicated no statistically significant difference between the model’s predictions and actual outcomes (*χ*^2^ = 6.939, *p* = 0.543, *p* > 0.05). The area under the ROC curve (AUC) was 0.875, with a sensitivity of 0.631 and a specificity of 0.891.

**Conclusion:**

The incidence of dysphagia in hospitalized elderly patients with frailty is relatively high. The main influencing factors include age, history of coughing, polypharmacy, malnutrition, oral health assessment index, and oral health-related self-efficacy. Healthcare professionals should enhance their awareness of risk warning, conduct early screening, and implement preventive measures. The constructed risk prediction model demonstrates high calibration and discrimination abilities, providing a valuable reference for the early detection, prevention, and intervention of dysphagia in hospitalized elderly patients with frailty.

## 1 Introduction

Frailty is a common geriatric syndrome characterized not only by the decline of physical function but also by increased vulnerability to multiple systemic diseases. It significantly raises the risk of morbidity and mortality among older adults and contributes to reduced functional capacity and quality of life (1, 2). Studies have shown that impaired muscle function in frail older adults often involves the swallowing muscles, leading to dysphagia. Dysphagia, in turn, impairs nutritional intake and increases the risk of aspiration pneumonia and choking, which can further worsen frailty, creating a vicious cycle that poses serious challenges for clinical management and care (3, 4).

International studies report that up to 70.6% of pre-frail individuals over the age of 85 experience dysphagia (5, 6). In China, the prevalence of dysphagia among hospitalized frail and pre-frail older adults is 39.1 and 29.7%, respectively, while in community-dwelling frail older adults it reaches 25.82% (7, 8). The occurrence of dysphagia is influenced by a variety of factors, including physiological decline, comorbidities, pharmacological treatments, and psychosocial conditions (9). Accurately identifying these factors is critical for developing personalized care plans and implementing effective early interventions.

However, current research has primarily focused on disease-related dysphagia, such as that caused by stroke or Parkinson’s disease (10), while limited attention has been given to swallowing dysfunction arising from natural aging processes. Moreover, comprehensive analyses of the contributing factors in frail populations remain scarce, and validated risk prediction tools are lacking. This hampers clinicians’ ability to recognize high-risk individuals in a timely manner.

To address this gap, the present study analyzed clinical data from hospitalized frail older adults to identify key risk factors associated with dysphagia and to develop a multidimensional risk prediction model. This model aims to enhance the precision of early screening, reduce dysphagia-related complications, and ultimately improve patient outcomes. The findings offer an evidence-based reference for healthcare professionals to support clinical decision-making and inform the development of more effective prevention and management strategies for frail older adults, contributing to the advancement of geriatric care systems.

## 2 Materials and methods

### 2.1 General information

#### 2.1.1 Inclusion and exclusion criteria

A total of 300 frail older inpatients admitted to our hospital between May and December 2024 were recruited using convenience sampling. This study was approved by the Medical Ethics Committee of our hospital (Approval No. S20240242-01).

##### 2.1.1.1 Inclusion criteria

1. Age ≥65 years;
2. Assessed as frail or pre-frail according to the Fried Frailty Phenotype Scale (11);
3. Able to eat orally without the use of nasogastric tubes or gastrointestinal fistulas;
4. Medically stable and fully conscious;
5. Provided informed consent and voluntarily participated in the study.

##### 2.1.1.2 Exclusion criteria included

1. Dysphagia caused by other organic diseases;
2. Diagnosed with Alzheimer’s disease or other types of dementia;
3. Severe visual, hearing, or speech impairments that hinder communication.

#### 2.1.2 Assessment of dysphagia

**Figure 1:**
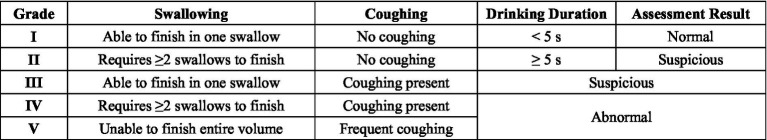
Kubota Water Swallow Test (WST) grading criteria.

Dysphagia was assessed using the Kubota Water Swallow Test (WST) (12), as illustrated in Figure 1. According to established literature (13), both “suspicious” and “abnormal” results were classified as indicative of dysphagia in this study.

### 2.2 Research methods

#### 2.2.1 Sample size estimation

According to the general rule for logistic regression, the required sample size should be at least 15–20 times the number of independent variables (14). In this study, 18 potential predictor variables were initially considered. Taking into account an anticipated attrition rate of 5–10%, the minimum sample size was preliminarily calculated as (18 × 15) × 1.05 = 284 cases. Considering the practical conditions of the research setting and the feasibility of the preliminary investigation, a stepwise backward likelihood ratio (backward LR) method was employed for variable selection during the model construction. The final model included six significant predictors: age, polypharmacy, history of choking, malnutrition, oral health status, and oral health-related self-efficacy. Ultimately, a total of 287 participants were enrolled, meeting the requirements for model stability.

#### 2.2.2 Study variables and instruments

Based on expert consultations and thematic discussions, 18 potential risk factors were identified. Details are as follows:

1. General Information Questionnaire: Including sex, age, living arrangement, place of residence, education level, pre-retirement occupation, marital status, smoking and drinking history, number of chronic diseases, polypharmacy, history of choking, and oral condition.
2. Nutritional Status: Assessed using the Mini Nutritional Assessment–Short Form (MNA-SF), which consists of six items with a total score of 14. A score of 11–14 indicates normal nutrition, and <11 indicates malnutrition. The tool has a sensitivity of 85.7% and specificity of 96% (15).
3. Sleep Quality: Evaluated using the Pittsburgh Sleep Quality Index (PSQI) (16, 17), with a total score of 21. Higher scores indicate poorer sleep quality.
4. Geriatric Oral Health Self-Efficacy: Measured using the Chinese version of the Geriatric Self -Efficacy Scale for Oral Health (GSEOH) translated by Xu et al. (18). The scale comprises 3 dimensions with 20 items, scored from 20 to 80. Higher scores reflect stronger oral health-related self-efficacy. The Cronbach’s *α* coefficient is 0.913.
5. Oral Health Status: Assessed using the Chinese version of the Geriatric Oral Health Assessment Index (GOHAI) translated by Ling et al. (19), consisting of 3 dimensions and 12 items, with a total score ranging from 12 to 60. The Cronbach’s *α* coefficient is 0.81.
6. Depression: Evaluated using the 5-item Geriatric Depression Scale (GDS-5), with a total score of 5. A score ≥2 indicates depression. The tool has a sensitivity of 94% and specificity of 81% (20).

#### 2.2.3 Data collection

A research team was established and trained prior to the survey. A pilot study was conducted from January to March 2024 with 30 participants to test and revise the questionnaire. Formal data collection was carried out between May and December 2024 through one-on-one interviews.

#### 2.2.4 Statistical analysis

Univariate analyses were conducted for each variable. Continuous data were described using mean ± standard deviation and compared using the *t*-test. Categorical data were described as frequencies and proportions and analyzed using the *χ*^2^ test. A *p*-value <0.05 was considered statistically significant. For multivariate analysis, variables were selected using backward likelihood ratio (backward LR) logistic regression. Model performance was evaluated using the Hosmer–Lemeshow goodness-of-fit test and the area under the Receiver Operating Characteristic (ROC) curve.

## 3 Results

### 3.1 Univariate analysis

Among the 287 hospitalized frail older adults included in this study, 103 (35.9%) were identified as having dysphagia. Among patients with a history of choking (*n* = 202), 80 (39.6%) had dysphagia, compared with 23 (27.1%) among those without a choking history (*n* = 85), with a statistically significant difference (*χ*^2^ = 4.092, *p* = 0.043).

Significant differences were observed between the dysphagia and non-dysphagia groups in terms of age, education level, smoking status, comorbidities, polypharmacy, nutritional status, choking history, oral health condition, oral health assessment score, oral health self-efficacy, and depressive symptoms (*p* < 0.05). See Table 1 for details.

**Table 1:** Univariate analysis of swallowing disorders in hospitalized elderly frailty patients.

Variable	Content	*n* (%)		Statistical metric	*p*
		Swallowing disorder group (*n* = 103)	Non-swallowing disorder group (*n* = 184)		
Age	<75 years	37 (35.9%)	92 (50.0%)	5.289^a^	0.021
	≥75 years	66 (64.1%)	92 (50.0%)		
Gender	Male	54 (52.4%)	85 (46.2%)	1.027^a^	0.311
	Female	49 (47.6%)	99 (53.8%)		
Education level	Illiterate	20 (22.3%)	15 (8.2%)	−3.457^c^	<0.001
	Primary school or below	37 (33.0%)	53 (26.6%)		
	Junior high school	29 (28.2%)	63 (34.2%)		
	Senior high school/Secondary vocational school	12 (11.7%)	35 (19.0%)		
	College or above	5 (4.9%)	18 (12.0%)		
Marital status	Married	76 (73.8%)	135 (73.4%)	0.006^a^	0.939
	Divorced or widowed	27 (26.2%)	49 (26.6%)		
Living arrangement	Not living alone	83 (80.6%)	157 (85.3%)	1.085^a^	0.298
	Living alone	20 (19.4%)	27 (14.7%)		
Occupation before retirement	Light physical labor	45 (43.7%)	87 (47.3%)	0.343^a^	0.558
	Heavy physical labor	58 (56.3%)	97 (52.7%)		
Residence location	Rural	71 (68.9%)	107 (58.2%)	3.258^a^	0.071
	Urban	32 (31.1%)	77 (41.8%)		
Smoking habit	Yes	59 (57.3%)	70 (38.0%)	9.877^a^	0.002
	No	44 (42.7%)	114 (62.0%)		
Alcohol consumption	Yes	34 (33.0%)	56 (30.4%)	0.203^a^	0.652
	No	69 (67.0%)	128 (69.6%)		
Multimorbidity	No	18 (17.5%)	65 (35.3%)	10.236^a^	0.001
	Yes	85 (82.5%)	119 (64.7%)		
Polypharmacy	No	40 (38.8%)	116 (63.0%)	15.598^a^	<0.001
	Yes	63 (61.2%)	68 (37.0%)		
History of coughing or choking	No	23 (22.3%)	62 (33.7%)	4.092^a^	0.043
	Yes	80 (77.7%)	122 (66.3%)		
Oral health status	No damage (repaired if damaged)	46 (44.7%)	108 (58.7%)	5.231^a^	0.022
	Unrepaired damage	57 (55.3%)	76 (41.3%)		
Malnutrition	No	70 (68.0%)	162 (88.0%)	17.191^a^	<0.001
	Yes	33 (33.0%)	22 (12.0%)		
Oral health self-efficacy score		56.02 ± 7.474	60.38 ± 4.884	5.964^b^	<0.001
Oral health evaluation index score		42.50 ± 5.312	47.54 ± 3.750	9.370^b^	<0.001
Sleep quality	Excellent	23 (22.3%)	36 (19.6%)	−0.063^c^	0.950
	Good	25 (24.3%)	52 (28.3%)		
	Average	34 (33.0%)	62 (33.7%)		
	Poor	21 (20.4%)	34 (18.5%)		
Depression	No	85 (82.5%)	173 (94.0%)	9.610^a^	0.002
	Yes	18 (17.5%)	11 (6.0%)		

a *X*^2^ value.

b *t* value.

c *z* value.

### 3.2 Multivariate logistic regression analysis

Eleven variables found to be significant in the univariate analysis were included as independent variables in the multivariate logistic regression. Dummy variables were created for categorical predictors. Variable coding is shown in Table 2, and dummy variable definitions in Table 3.

**Table 2:** Variable assignment for multivariate logistic regression analysis.

Variable type	Variable name	Assigned value
Dependent	Swallowing disorder	0 = No, 1 = Yes
Independent	Age	0 = <75 years, 1 = ≥75 years
	Education level	1 = Illiterate, 2 = Primary school or below, 3 = Junior high school, 4 = High school/technical secondary school, 5 = College or above
	Smoking	0 = No, 1 = Yes
	Multiple comorbidities	0 = No, 1 = Yes
	Polypharmacy	0 = No, 1 = Yes
	History of choking/coughing	0 = No, 1 = Yes
	Malnutrition	0 = No, 1 = Yes
	Oral and dental condition	1 = No missing teeth or missing teeth restored, 2 = Missing teeth not restored
	Depression	0 = No, 1 = Yes

**Table 3:** Dummy variable settings table.

Variable type		Frequency	Parameter coding			
			(1)	(2)	(3)	(4)
Educational level	Illiterate	38	1	0	0	0
	Primary school or below	83	0	1	0	0
	Junior high school	92	0	0	1	0
	Senior high school/Secondary vocational school	47	0	0	0	1
	College or above	27	0	0	0	0

The results indicated that age, history of choking, polypharmacy, malnutrition, oral health assessment score, and oral health self-efficacy were independently associated with dysphagia among hospitalized frail older adults (*p* < 0.05) (see Table 4).

**Table 4:** Multivariate logistic regression analysis of risk factors for swallowing disorder in hospitalized frail older adults.

Variable	B	SE	Wals	*p*	OR	95% CI	
						Lower	Upper
Age	0.770	0.336	5.262	0.022	2.160	1.119	4.170
Polypharmacy	0.919	0.329	7.789	0.005	2.506	1.315	4.779
History of choking	1.009	0.377	7.159	0.007	2.742	1.310	5.742
Malnutrition	1.208	0.413	8.542	0.003	3.348	1.489	7.529
Oral health-related self-efficacy score	−0.113	0.029	15.042	0.000	0.894	0.844	0.946
Oral health status evaluation index score	−0.262	0.045	33.787	0.000	0.770	0.705	0.841
Constant	10.200	3.023	11.383	0.001	0.000		

B, regression coefficient; SE, standard error; Wals, Wals chi-square test statistic; OR, odds ratio; CI, confidence interval.

### 3.3 Risk prediction model development and evaluation

#### 3.3.1 Construction and application of a nomogram prediction model

**Figure 2:**
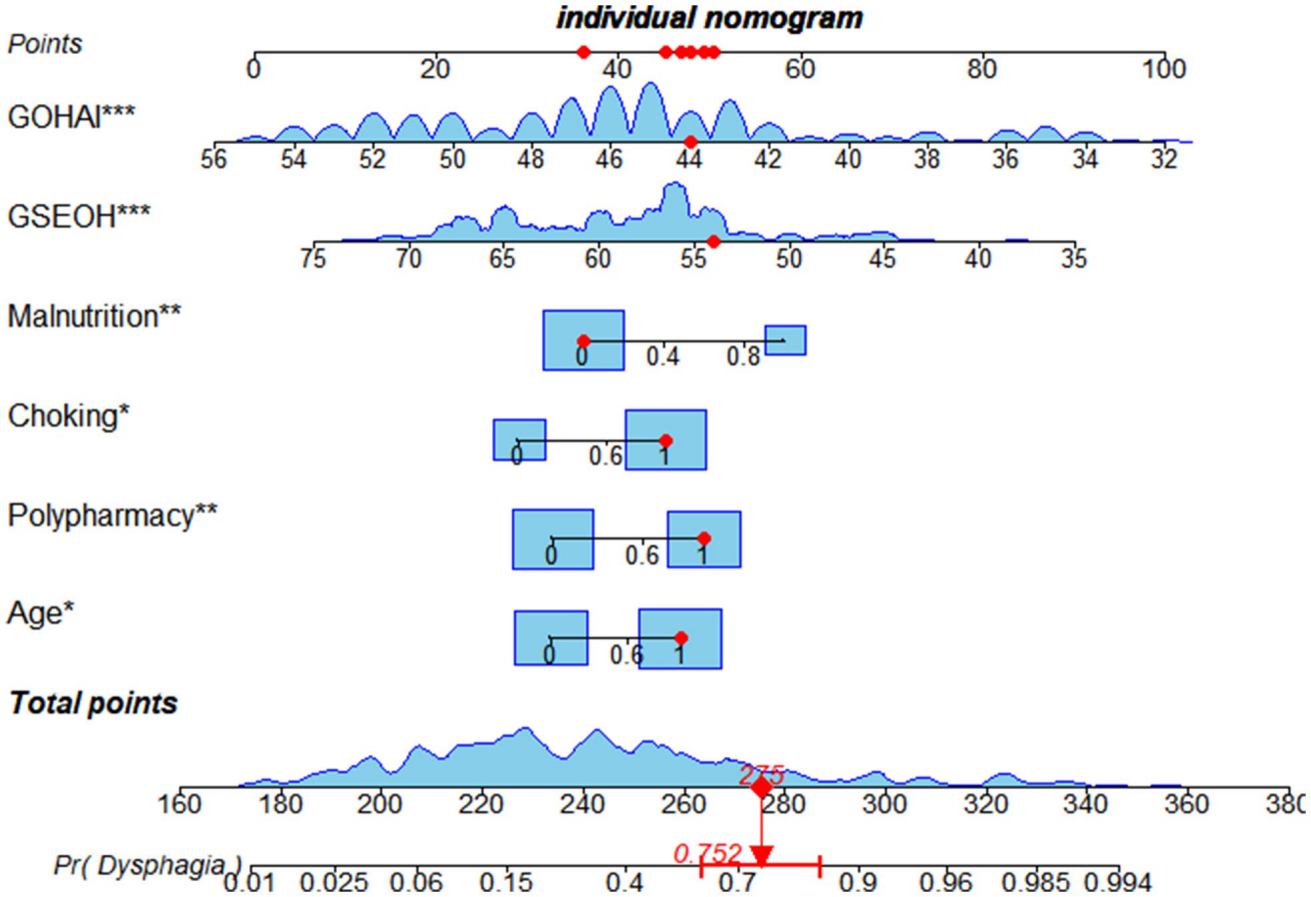
Nomogram of the risk prediction model.

Based on the results of multivariate logistic regression analysis, a risk prediction model for dysphagia in hospitalized elderly patients with frailty was developed as follows: Logit *p* = 0.770 × Age + 0.919 × Polypharmacy + 1.009 × History of Coughing + 1.208 × Malnutrition − 0.113 × Oral Health-Related Self-Efficacy − 0.262 × Oral Health Assessment Index + 10.200. To enhance the clinical utility of the model, the optimal cut-off value was determined to be 0.426 based on the ROC curve analysis combined with the Youden Index method. Patients were stratified into three risk groups accordingly: Low-risk group (*p* ≤ 0.2): Routine care is recommended, with monthly follow-up; Moderate-risk group (0.2 < *p* ≤ 0.5): Bedside swallowing screening and individualized dietary guidance are advised; High-risk group (*p* > 0.5): Further assessment with videofluoroscopic swallowing study (VFSS) or fiber optic endoscopic evaluation of swallowing (FEES) should be performed as soon as possible, followed by systematic rehabilitation interventions. In addition, a nomogram was developed to facilitate clinical application, allowing healthcare providers to perform rapid and individualized risk assessment based on specific patient characteristics (see Figure 2).

#### 3.3.2 Model evaluation

**Figure 3:**
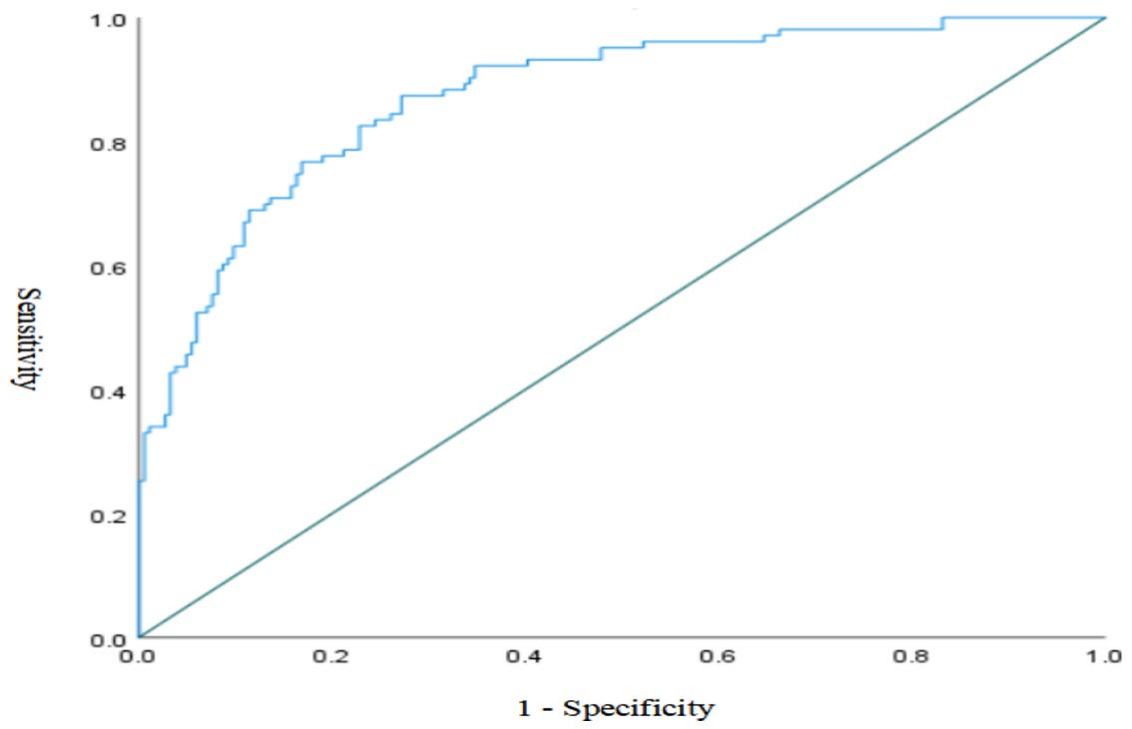
ROC curve of the risk prediction model.

1. Calibration: The calibration of the prediction model was assessed using the Hosmer–Lemeshow (H–L) goodness-of-fit test. The test yielded a *χ*^2^ value of 6.939 with a *p*-value of 0.543 (*p* > 0.05), indicating no significant difference between the predicted and observed outcomes. This suggests that the model has good calibration.
2. Discrimination: To evaluate the discriminatory ability of the model, a Receiver Operating Characteristic (ROC) curve was plotted with 1-specificity on the *X*-axis and sensitivity on the *Y*-axis. The area under the curve (AUC) was 0.875 (95% CI, 0.833 ~ 0.916, *p* < 0.001), which indicates excellent discriminative performance. See Figure 3.

#### 3.3.3 Model validation

**Figure 4:**
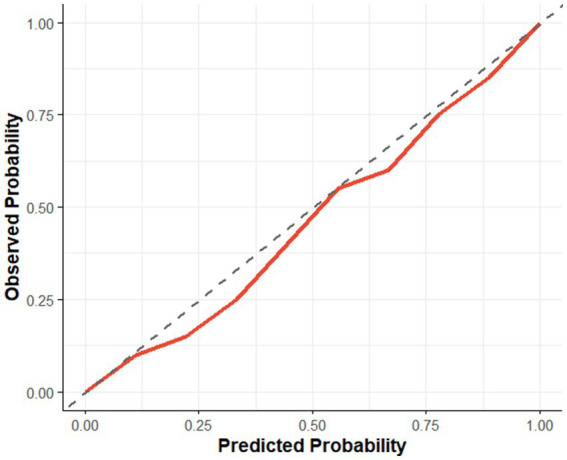
Calibration curve.

To evaluate the internal validity and predictive performance of the logistic regression model, bootstrap resampling (*B* = 100) was performed. The model yielded a *C*-index of 0.84, indicating good discriminative ability. The calibration slope was 0.93 and the intercept was −0.035, with a maximum calibration error (Emax) of 0.022, suggesting good agreement between predicted probabilities and observed outcomes. The Brier score was 0.151, indicating an acceptable level of overall prediction error. Additionally, the model’s mean absolute error (MAE) was 0.018, mean squared error (MSE) was 0.0004, and the 90th percentile of absolute error was 0.026, reflecting minimal prediction bias and good overall calibration accuracy (see Figure 4).

## 4 Discussion

In this study, 103 out of 287 hospitalized frail older adults were identified with dysphagia, indicating a prevalence rate of 35.9%. This suggests that dysphagia is relatively common among frail elderly inpatients. Previous studies have reported similar rates: Wang et al. (21) found a prevalence of 39.1%, and Ren et al. (22) reported a rate of 49.2%. Our findings indicate that dysphagia in this population is influenced by multiple factors—including age, malnutrition, and polypharmacy—which are often underrecognized in clinical practice. Therefore, it is crucial to enhance early identification and screening for dysphagia among frail older adults and to implement targeted preventive interventions.

This study identified age as a significant risk factor for dysphagia, aligning with the findings of Zeng et al. (23). With aging, physiological changes such as pharyngeal muscle degeneration, reduced hyoid and laryngeal mobility, and delayed swallowing reflexes occur, all of which increase the risk of dysphagia (24). Additionally, age-related decline in neurological control further impairs the coordination of swallowing, heightening the risk of aspiration (25). Older adults may also have diminished awareness of their swallowing difficulties, leading to underreporting and delayed intervention. Thus, early assessment and intervention—such as swallowing rehabilitation and dietary modifications—should be prioritized for elderly patients, especially those of advanced age.

A history of choking was found to be a strong predictor of dysphagia in this study. This symptom reflects impaired airway protection or delayed swallowing reflex. Logistic regression analysis confirmed it as an independent risk factor (OR = 2.742, *p* = 0.007), consistent with findings by Jia et al. (26). Central nervous system degeneration in frail older adults may delay swallowing reflex initiation, allowing food to remain in the pharynx and increasing the risk of aspiration. In addition, reduced muscle mass and poor glottic closure weaken airway protection. Repeated choking episodes may also lead to fear of eating, resulting in nutritional decline and a vicious cycle of worsening frailty and dysphagia (27). Therefore, a history of choking should serve as an early warning sign for potential dysphagia, warranting further assessment or objective testing. Interventions such as swallowing training, dietary adjustments, and enhanced oral care should be implemented to reduce aspiration-related complications and improve quality of life.

Polypharmacy was another significant risk factor identified in our study. Frail older adults who take multiple medications have an elevated risk of dysphagia. Several drugs, including sedatives and antidepressants, can impair swallowing reflexes, while anticholinergics may reduce saliva production, increasing the risk of dry mouth and impaired mastication. Drug interactions may further contribute to swallowing difficulties. Clinicians should carefully evaluate medication necessity, reduce polypharmacy when possible, and monitor the effects of medications on swallowing function to minimize related complications.

Malnutrition was found to be a major contributor to dysphagia, in line with the findings of Tagliaferri et al. (28). Nutritional deficits lead to muscle atrophy, including muscles involved in swallowing, resulting in decreased swallowing strength and coordination (29). Deficiencies in key nutrients, such as vitamin B12 and vitamin D, may impair neuromuscular function and accelerate decline in swallowing ability. Thus, early nutritional screening and timely intervention are critical. For frail older adults at risk of malnutrition, nutritional support—including increased intake of high-quality protein and micronutrients—should be emphasized to maintain swallowing-related muscle function.

Our results show that poor oral health status, as indicated by lower scores on the oral health assessment index, is associated with a higher risk of dysphagia. Common oral issues such as tooth loss, caries, periodontal disease, and xerostomia can impair chewing efficiency and food processing, thereby increasing swallowing difficulty (30). Furthermore, oral microbiota imbalance may cause local inflammation, further affecting swallowing. Therefore, it is essential to promote regular oral hygiene practices and routine dental assessments among frail older adults to reduce the risk of dysphagia.

Oral health-related self-efficacy also emerged as a key influencing factor. Self-efficacy reflects an individual’s belief in their ability to manage health behaviors. Lower oral health self-efficacy may result in neglect of oral hygiene and reduced adherence to oral care routines, thereby increasing the risk of oral diseases and indirectly affecting swallowing function. Studies have shown (31) that improving oral health self-efficacy enhances proactive participation in oral hygiene, which in turn reduces the risk of dysphagia. Health education, personalized counseling, and behavioral interventions can be used to improve older adults’ confidence and ability to manage oral health.

Limitations and Future Directions: Although this study successfully identified six independent risk factors for dysphagia among hospitalized elderly patients with frailty and developed a risk prediction model with good discrimination and calibration, several limitations should be acknowledged. First, unmeasured confounding may have influenced the results. Although the study incorporated variables related to demographics, medical history, nutrition, oral health, and psychological status, other potentially important factors were not collected or analyzed. These include subclinical neurological impairments (e.g., silent stroke, mild cognitive impairment), specific indicators of functional status such as muscle strength or sarcopenia, the severity of comorbid conditions (rather than just the count), details on specific medications (e.g., anticholinergics, sedatives) that may differently affect swallowing, and information on whether participants had received prior swallowing rehabilitation. The absence of these factors may introduce residual confounding, potentially biasing the estimated effects of included variables and limiting the model’s accuracy and generalizability. Second, the use of convenience sampling in a single tertiary hospital may have led to several forms of bias. The sample may not represent the broader frail elderly population, as patients with severe illness or communication difficulties were excluded (selection bias), and patients in tertiary care settings often differ from those in community or primary care settings (referral bias). Furthermore, participants who agreed to be included in the study may have had higher health awareness or different behavior patterns compared to non-participants (volunteer bias), which may have affected relevant variables such as self-efficacy or oral hygiene practices. These biases could result in model overfitting, reduced external validity, and calibration drift when applied to other populations. To enhance the robustness and applicability of the model, future research should consider conducting multicenter studies using random or stratified sampling methods, include a more comprehensive range of risk factors (e.g., neurological function, sarcopenia, specific medication classes), and perform external validation. Moreover, further efforts are warranted to explore more precise prevention and intervention strategies, ultimately improving the clinical utility of dysphagia risk prediction models for frail elderly patients.

## Data Availability

The original contributions presented in the study are included in the article/supplementary material, further inquiries can be directed to the corresponding author.
